# Impact of the SARS-CoV-2 Delta Variant on the Psychological States and Health-Related Quality of Life in Patients With Crohn’s Disease

**DOI:** 10.3389/fmed.2022.795889

**Published:** 2022-03-29

**Authors:** Jiajia Li, Yunyun Sun, Xiaolin Hu, Tiantian Zhao, Guanghuai Yao, Weiming Xiao, Yanbing Ding, Sicong Hou, Mei Wang

**Affiliations:** Department of Gastroenterology, Affiliated Hospital of Yangzhou University, Yangzhou, China

**Keywords:** COVID-19, SARS-CoV-2 Delta variant, Crohn’s disease, mental health, health-related quality of life

## Abstract

**Background:**

Since the outbreak of the coronavirus disease 2019 (COVID-19) pandemic first reported in Wuhan, China, several research on the psychological impact of the pandemic on patients with Crohn’s disease (CD) have been conducted. However, with the progression of the global pandemic and the emergence of the SARS-CoV-2 B.1.617.2 (Delta) variant, follow-up studies need to be performed to monitor the alterations of psychological status and health-related quality of life (HRQoL) among CD patients.

**Aims:**

We aimed to evaluate the impact of the SARS-CoV-2 Delta variant on the mental health and life quality among the CD population and tried to explore potent risk factors.

**Methods:**

This observational study included 153 CD patients who responded to our pre-designed self-reported questionnaire. Demographic, clinical, and psychological information were collected and analyzed.

**Results:**

Quite a number of CD patients were confronted with different levels of anxiety and depression, with incidence of 28.10 and 31.37% for anxiety and depression, respectively. Compared with non-pandemic circumstances, the life quality of CD patients due to the present situation was more often compromised. Isolation [odds ratio (OR): 4.71, *P* = 0.007] was verified as a risk factor for anxiety while use of telemedicine could help relieve anxiety (OR: 0.22, *P* < 0.001). Worsening of symptoms (OR: 4.92, *P* = 0.006), isolation (OR: 5.75, *P* = 0.005), and drug withdrawn (OR: 2.66, *P* = 0.026) were identified to be independent factors for developing depression. Likewise, use of telemedicine (OR: 0.13, *P* < 0.001) was negatively related to depression. Considering life quality, vaccination (OR: 3.07, *P* = 0.021) together with no medication (OR: 7.73, *P* = 0.010) was relevant to better life quality while worsening of symptoms (OR: 0.09, *P* = 0.034) were an independent risk factor for impaired life quality.

**Conclusion:**

Many CD patients suffered from symptoms of anxiety and depression and impaired life quality during the COVID-19 pandemic. Those in isolation or with worsening of symptoms and drug withdrawn were more prone to experience psychological stress. Individualized management such as drug delivery and telemedicine should be promoted to maintain control of mental health and life quality during the pandemic.

## Introduction

Since the outbreak of the coronavirus disease 2019 (COVID-19) pandemic first reported in Wuhan, China, numerous variants of concern (VoCs) of SARS-CoV-2 have been revealed, among which the Delta variant (B.1.617.2) is by far the dominant strain (1–3). First detected in India in December 2020, the Delta variant has now been detected nearly all of the globe (4). The Delta variant is characterized by mutations of the spike proteins such as T19R, T478K, Δ157-158, P681R, L452R, D614G, D950N, etc. (5). Several of these mutations may influence immune responses against the key antigenic regions of receptor-binding protein and deletion of part of the N-terminal domain while some mutations at the S1–S2 cleavage site appear to be associated with increased replication, which leads to higher viral loads and increased transmission (6, 7). It has been reported that the relative viral loads of the Delta variant of quarantine contact cases was significantly higher than the original lineage (8–10). With a remarkably elevated transmissibility (40–60% higher compared with the Alpha variant), the Delta variant has exerted a disastrous impact on the infection and mortality rates globally (11–13). In China, the Delta variant accounts for most of the new cases since the year of 2021. Previous research has implicated that the COVID-19 pandemic is likely to cause abnormality of emotion, cognition, behavior and quality of life, especially in population with chronic diseases such as systemic lupus erythematosus, rheumatoid arthritis and inflammatory bowel disease (IBD), in whom psychological distress and somatic diseases could influence mutually (14–16).

Crohn’s disease (CD) is a subtype of IBD, which is featured by chronic and relapsing inflammation of the gastrointestinal tract (17). The etiology and pathogenesis of CD still remain unclear. It is commonly recognized that the interplay among genetic background, environment triggers, host microbiota, and immune response contributes to the initiation of CD (18). Importantly, due to the chronic and recurrent behavior of CD, psychological distress also plays an indispensable role throughout the disease course (19). Anxiety and depression are very common in CD patients, with a morbidity of anxiety and/or depression about 29–35% in the remission stage and this rate can rise to as high as 60–80% during relapses (20). Compared with the general population, the rate of psychological disorders such as anxiety and depression in CD patients, especially in the active stage, is 2–3 times higher (21). In turn, Frolkis et al. reported that depression is related to early clinical recurrence and disease severity in CD, which could be mitigated by treatment of depression (22). Several mechanisms underlying the psychological disorders in CD patients have been revealed, among which the theory of the brain-gut axis is the most wildly acknowledged. Briefly, there are abundant autonomic nervous plexus connections between the enteric nervous system (ENS) and central nervous system, which is also known as brain-gut axis. On one hand, the motility, sensory and secretory functions and pain thresholds of the gastrointestinal tract can be directly or indirectly affected by psychological and emotional stress through the brain-gut axis. In this process, substance P (SP), vasoactive intestinal peptides (VIP), various neuropeptides, neurotransmitters and hormones play a part. On the other hand, intestinal inflammation can also act on the central system by the production of pro-inflammatory cytokines such as tumor necrosis factor α (TNF-α), thus inducing the symptoms of anxiety or depression (23, 24).

Apart from psychological status, health-related quality of life (HRQoL) has become another concern in the management of CD patients (25). HRQoL is often compromised in CD patients especially during disease relapses (26). Additionally, factors independent of disease activity also contribute to the alteration of life quality (27). A recent study demonstrated that during the COVID-19 pandemic, HRQoL among CD patients was impaired but the underlying variables still awaits exploration (28).

In the current situation where social isolation and uncertainty are likely to occur, CD patients are facing an increasing risk of suffering from worsening anxiety and depression, which may cause relapse or escalation of CD activity. Previous studies conducted at the beginning of the pandemic suggested that COVID-19 exerted negative effects on psychological and disease outcomes among CD patients (28, 29). However, with the progression of the global pandemic and the emergence of the SARS-CoV-2 Delta variant, there have been changes in how patients with CD perceive and respond to the pandemic. For example, many patients have mastered protection skills against the virus and might have been vaccinated; some patients have learned how to manage CD under the pandemic, while another group of patients are more concerned of the Delta variant. These changes could lead to alterations in the psychological states and HRQoL under this kind of situation. Therefore, in this study, we aimed to evaluate the impact of the SARS-CoV-2 Delta variant on the mental health and life quality among CD population and tried to explore potent risk factors, so as to modify variables that are feasible to intervention and set optimized management for susceptible CD patients.

## Materials and Methods

### Design and Setting

This study is a cross-sectional, observational analysis using information of CD patients diagnosed and treated at Affiliated Hospital of Yangzhou University from 2012 to 2021. The study was conducted between 25 August and 15 September 2021. All protocols in the study were carried out in accordance with the principles of the Declaration of Helsinki. This study was approved by the Institutional Review Board and Ethics Committee of Affiliated Hospital of Yangzhou University (REC ref 2021-YKL06-09-006). Completion of the study questionnaire was regarded as informed consent.

### Patients

Clinical staff at Department of Gastroenterology, Affiliated Hospital of Yangzhou University first screened potential participants that met the inclusion criteria of this study. Inclusion criteria consisted of (1) diagnosis with CD based on the criteria determined by the European Crohn’s and Colitis Organization (ECCO) guidelines (30, 31) and (2) age >18 years. Exclusion criteria were comprised of the following: (1) history of mental diseases diagnosed prior to CD onset (e.g., mood disorders, schizophrenia, psychosis, obsessive-compulsive disorder, psychoactive substance abuse, post-traumatic or acute stress-disorder, and intellectual disability); (2) accepting pharmacological or psychological treatment for mental health problems at the time of study recruitment; (3) diagnosed with somatic diseases reported to have an impact on the psychological state (e.g., diabetes mellitus, thyroid dysfunction, heart failure, and renal insufficiency). Such patients were excluded for these conditions are potent confounding variables for psychological assessment. Also, those lacking sufficient data for our study were ruled out.

### Measures

After recruitment of eligible patients, a pre-designed self-reported questionnaire was sent *via* an online survey platform. The questionnaire consists of four sections. The first section collects basic demographic and socioeconomical information of the CD patients including age, gender, education level, occupation, income level, etc. The second section focuses on the clinical characteristics of CD such as age at the time of the diagnosis, disease duration, involvement of perianal disease, presence of extraintestinal manifestations, history of CD related surgeries, medication, and current symptoms (especially worsening of symptoms). For each patient, the Harvey-Bradshaw Disease Activity Index (HBI) for CD was calculated by experienced gastroenterologists to assess the disease severity. A score of ≥5 was defined as existence of disease activity.

The third section assesses the psychological state and HRQoL of the participants. The Generalized Anxiety Disorder Scale 7 (GAD-7) was used to assess the frequency of the patients’ anxiety in the past 2 weeks. It consists of seven questions and each question has four choices on a scale of 0 to 3, totaling 21 points. The higher the score is, the more serious the anxiety degree is. A score 0–4 points is within the normal range; 5–9 points indicate mild anxiety levels; 10–14 points indicate moderate anxiety levels while individuals scoring 15–21 points are considered to suffer from severe anxiety (32). With regards to depression, Patient Health Questionnaire-9 (PHQ-9) which is composed of nine depression-related items was adopted. Each item is scored on a scale of 0 to 3, with an overall score of 0 to 27. According to the score of PHQ-9, depression can be divided into five severity categories: minimal (0–4), mild (5–9), moderate (10–14), moderately severe (15–19), and severe (20–27) (33). To evaluate the HRQoL of the participants, the Inflammatory Bowel Disease Questionnaire (IBDQ) was applied (34). A total of 32 questions were included which reflected the intestinal symptoms, systemic symptoms, emotional functions, and social functions of the patients. The total score ranges from 32 to 224, with a higher score indicating a better quality of life.

The fourth section of our questionnaire concerns issues around COVID-19 and the SARS-CoV-2 Delta variant and how patient care is influenced. To assess the participants’ knowledge of the COVID-19 pandemic and the SARS-CoV-2 Delta variant, we designed nine questions related to the virus (Supplementary Table 1). With 1 point for each correct answer, the total score ranges from 0 to 9. Additionally, questions regarding the isolation status, drug withdrawn and vaccination are also included.

It was stressed that results the participants filled in would be made of the biggest value if they could give honest answers. Data extraction from the questionnaires was performed by two independent researchers for further analysis. In case of a dispute in the interpretation of the questionnaires, discussion among experienced experts in our department would be held to reach a consensus.

### Statistical Analysis

Statistical analysis was performed with SPSS version 22.0 (IBM SPSS Statistics, United States) and GraphPad Prism 5 (GraphPad Software, United States). Continuous variables were expressed as mean ± standard deviation (SD) while categorical variables are presented as proportions. To detect potent factors underlying altered psychological stress and life quality, binary logistic regression was performed. In the univariable analysis, candidate predictors were screened with a criterion of *P* ≤ 0.10. Then multivariable analysis was applied for further exploration to eliminate variables without statistical significance. A *P*-value < 0.05 was considered statistically significant in the multivariable analysis. The odds ratio (OR) was used as a measure of association between the variables and results of the psychological stress and life quality.

## Results

### Demographic Characteristics

As shown in Supplementary Figure 1, a total of 202 individuals responded to our questionnaire, of whom 49 were excluded from our analysis. Among these being excluded, 10 had a non-CD diagnosis; 23 previously suffered or were suffering from mental diseases at the time of our study; 8 were concomitant with other somatic diseases related to the psychological state, and the remaining 8 did not provide sufficient information. Basic characteristics are displayed in Table 1. The mean age of CD patients is 38.44 ± 13.00 years old, and the male: female ratio was 85:68. Sixty-nine percent were married, 27.45% single, and 3.92% divorced. In regard to the occupation: 20.92% were manual worker, 37.25% mental worker, 17.00% unemployed, 9.15% retired, and 15.69% student, and 18.90% retired. As for education level, 32.03% got a bachelor’s degree or higher. Eighty percent were covered by medical insurance, and smokers and drinkers accounted for 17.95 and 11.54%, respectively.

**TABLE 1:** Demographic characteristics of CD patients included in the study.

Variables	Mean ± SD or *n* (%)
Gender, *n* (%)	
Male	85 (55.56%)
Female	68 (44.44%)
Age, years (mean ± SD)	38.44 ± 13.00
Marital status, *n* (%)	
Married	105 (68.63%)
Single	42 (27.45%)
Divorced	6 (3.92%)
Occupation, *n* (%)	
Manual worker	32 (20.92%)
Mental worker	57 (37.25%)
Unemployed	26 (17.00%)
Retired	14 (9.15%)
Student	24 (15.69%)
Education level, *n* (%)	
Elementary	11 (7.19%)
High school	93 (60.78%)
Bachelor	44 (28.76%)
Post graduate	5 (3.27%)
Socio-economic status, *n* (%)	
Low	83 (54.25%)
Middle	32 (20.92%)
High	38 (24.84%)
Medical insurance coverage, *n* (%)	123 (80.39%)
Smoking	28 (17.95%)
Drinking	18 (11.54%)

*SD, standard deviation.*

### Disease Characteristics and Life Changes Caused by the SARS-CoV-2 Delta Variant

The mean disease duration of CD patients is 5.37 ± 4.81 years (Table 2). According to the HBI score, 113 patients were sorted into the inactive group, while the rest 40 were classified as having disease activity (33 moderate and 14 severe). Concerning the IBD medication, 15 patients (9.80%) had not received prior medication, and the number of patients using 5-aminosalicylic acid (5-ASA), prednisolone, immunomodulators, biologics and traditional Chinese medicine is 31 (20.26%), 3 (1.96%), 56 (36.60%), 80 (52.29%), and 3 (1.96%), respectively. A total of 34 patients (22.22%) received surgical treatment. In 81 patients (52.94%), perianal involvement was reported.

**TABLE 2:** Disease characteristics and the SARS-CoV-2 Delta variant-related items.

Variables	Mean ± SD or *n* (%)
Disease duration, years (mean ± SD)	5.37 ± 4.81
Disease activity, *n* (%)	
Inactive (HBI < 5)	113 (75.16%)
Moderate active (5 ≤ HBI < 9)	33 (21.57%)
Severe active (HBI ≥ 9)	7 (4.58%)
Perianal involvement, *n* (%)	81 (52.94%)
History of surgery, *n* (%)	34 (22.22%)
Treatment, *n* (%)	
5-ASA	31 (20.26%)
Prednisolone	3 (1.96%)
Immunomodulators	56 (36.60%)
Biologics	80 (52.29%)
Traditional Chinese medicine	3 (1.96%)
No medication	15 (9.80%)
Isolation, *n* (%)	20 (13.07%)
Use of telemedicine, *n* (%)	86 (56.21%)
Vaccination, *n* (%)	55 (35.95%)
Knowledge of the Delta variant	7.46 ± 1.40
Drug withdrawn, *n* (%)	64 (41.83%)
Worsening of symptoms, *n* (%)	44 (28.76%)
Fear of contracting COVID-19, *n* (%)	62 (40.53%)
Stopped working, *n* (%)	41 (26.80%)
Willingness to turn to psychological help	21 (13.73%)

*SD, standard deviation; HBI, Harvey-Bradshaw Disease Activity Index; 5-ASA, 5-aminosalicylic acid; COVID-19, coronavirus disease 2019.*

**FIGURE 1:**
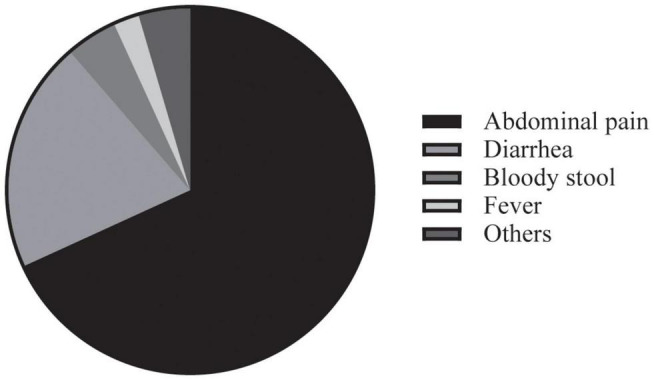
Worsening symptoms during the COVID-19 pandemic.

**FIGURE 2:**
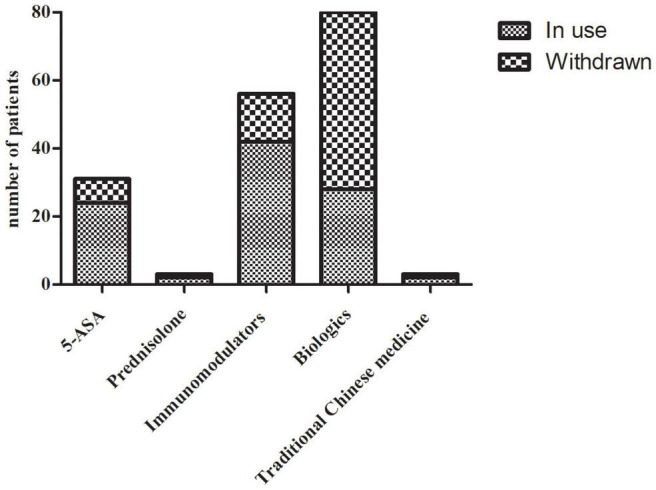
Rates of drug withdrawn for different medications. 5-ASA, 5-aminosalicylic acid.

During the lockdown, 44 patients (28.76%) experienced worsening of symptoms. Of those with exacerbated symptoms, 68% reported abdominal pain, and the rest mainly complained of diarrhea, bloody stool, fever, etc. (Figure 1). None of the included patients were infected with COVID-19, but 20 (13.07%) were isolated as close contacts. The shutdown led to drug withdrawn in 64 patients (41.83%) due to restricted accessibility to the hospital and pharmacies. Rates of drug withdrawn for different medications were shown in Figure 2. Additionally, to minimize the spread of SARS-CoV-2, IBD physicians in our institute tried to provide medical service *via* telemedicine, and this was utilized by 86 patients (56.21%). Owing to the strong advocation of the health authorities, 55 (35.95%) CD patients finished the COVID-19 vaccination, and the SARS-CoV-2 Delta variant is well known to CD patients (7.46 ± 1.40).

### Anxiety, Depression, and Health-Related Quality of Life Among Crohn’s Disease Patients During the SARS-CoV-2 Delta Variant Predominance

Using 5 as the cut-off value for the GAD-7 scale, the incidence of anxiety was 28.1% (43/153), with 34 in the mild group and 9 in the moderate group (Table 3). In terms of depression, 48 (31.37%) scored ≥5 for the PHQ-9 scale, which is regarded as the existence of depression, and among these, 36 (23.53%) presented mild depression, 10 (6.54%) moderate, and 2 (1.31%) moderately severe. During the closure, most of the CD patients (80.39%) maintained a good life quality (IBDQ score ≥209), while HRQoL of 30 patients (19.61%) was impaired. Among the various sources of psychological stress, drug withdrawn was the problem that bothers CD patients the most (41.83%), followed by fear of contracting SARS-CoV-2 Delta variant (62, 40.53%), worsening of symptoms (44, 28.76%) and suspension of work (41, 26.80%). During the pandemic, many cities in China have opened psychological counseling hotlines during the epidemic, but only a small proportion of our patients are aware of and are willing to turn to it for help (21, 13.73%).

**TABLE 3:** Level of anxiety, depression, and HRQoL among CD patients.

Variables	*n* (%)	Mean ± SD
Anxiety (GAD-7)		
No	110 (71.90%)	0.83 ± 1.20
Mild	34 (22.22%)	6.74 ± 1.31
Moderate	9(5.88%)	12.22 ± 1.64
Severe	0	/
Depression (PHQ-9)		
No	105 (68.63%)	1.24 ± 1.42
Mild	36 (23.53%)	7.17 ± 1.66
Moderate	10 (6.54%)	11.40 ± 1.58
Moderately severe	2 (1.31%)	17.00 ± 1.41
Severe	0	/
HRQoL (IBDQ)		
Low	123 (80.39%)	181.16 ± 22.00
High	30 (19.61%)	215.13 ± 4.60

*SD, standard deviation; GAD-7, Generalized Anxiety Disorder Scale 7; PHQ-9, Patient Health Questionnaire-9; HRQoL, health-related quality of life; IBDQ, Inflammatory Bowel Disease Questionnaire.*

### Factors Associated With Anxiety, Depression, and Health-Related Quality of Life Among Crohn’s Disease Patients During the SARS-CoV-2 Delta Variant Predominance

To explore the factors associated with anxiety in CD patients, we first applied univariable logistic regression to screen for potential variables. As shown in Table 4, a post graduate degree (OR: 40 [95% CI: 1.98–807.10], *P* = 0.016), higher disease activity (OR: 1.17 [95% CI: 1.02–1.34], *P* = 0.030), worsening of symptoms (OR: 2.69 [95% CI: 1.27–5.69], *P* = 0.010), isolation (OR: 4.94 [95% CI: 1.85–13.16], *P* = 0.001), better knowledge of the Delta variant (OR: 1.38 [95% CI: 1.03–1.85], *P* = 0.031) and drug withdrawn (OR: 1.94 [95% CI: 0.95–3.95], *P* = 0.070) were positively related to the occurrence of anxiety, while utility of telemedicine (OR: 0.26 [95% CI: 0.12–0.54], *P* < 0.001) was associated with less anxiety. After adjustment by multivariant analysis, isolation (OR: 4.71 [95% CI: 1.54–14.44], *P* = 0.007) was verified as a risk factor for anxiety while telemedicine could help relieve anxiety (OR: 0.22 [95% CI: 0.09–0.52], *P* < 0.001).

**TABLE 4:** Factors associated with elevated anxiety.

Variables	Univariable analysis			Multivariable analysis		
	*P*	OR	95% CI	*P*	OR	95% CI
Gender (female)	0.445	0.76	0.37–1.55			
Age	0.932	1.00	0.97–1.03			
Marital status, *n* (%)						
Married						
Single	0.163	0.54	0.22–1.29			
Divorced	0.328	2.28	0.44–11.92			
Occupation, *n* (%)						
Manual worker						
Mental worker	0.612	1.28	0.50–3.30			
Unemployed	0.596	1.35	0.44–4.13			
Retired	0.320	0.43	0.08–2.29			
Student	0.320	0.51	0.14–1.92			
Education level						
Elementary						
High school	0.269	3.29	0.40–27.07	0.522	2.05	0.23–18.59
Bachelor	0.134	5.17	0.60–44.32	0.456	2.37	0.25–23.00
Post graduate	0.016*	40	1.98–807.10	0.132	10.96	0.49–247.00
Socio-economic status						
Low						
Middle	0.675	0.82	0.32–2.08			
High	0.997	1.00	0.43–2.34			
Medical insurance coverage	0.518	1.36	0.54–3.45			
Smoking	0.952	1.03	0.42–2.55			
Drinking	0.600	1.32	0.46–3.79			
Disease duration	0.414	1.03	0.96–1.11			
Disease activity (HBI)	0.030*	1.17	1.02–1.34	0.770	1.03	0.85–1.25
Perianal involvement	0.525	1.26	0.62–2.55			
History of surgery	0.139	1.84	0.82–4.11			
Treatment						
5-ASA	0.750	0.87	0.35–2.12			
Prednisolone	0.999	0	/			
Immunomodulators	0.922	1.04	0.50–2.15			
Biologics	0.207	1.59	0.78–3.25			
Traditional Chinese medicine	0.839	1.29	0.11–14.56			
No medication	0.466	0.61	0.16–2.29			
Isolation	0.001*	4.94	1.85–13.16	0.007**	4.71	1.54–14.44
Use of telemedicine	<0.001*	0.26	0.12–0.54	0.001**	0.22	0.09–0.52
Vaccination	0.324	1.43	0.70–2.91			
Knowledge of the Delta variant	0.031*	1.38	1.03–1.85	0.156	1.27	0.91–1.78
Drug withdrawn	0.070*	1.94	0.95–3.95	0.291	1.58	0.68–3.69
Worsening of symptoms	0.010*	2.69	1.27–5.69	0.405	1.59	0.53–4.73
Fear of contracting COVID-19	0.958	0.98	0.48–2.02			
Stopped working	0.713	0.83	0.31–2.25			

*OR, odds ratio; CI, confidence interval; HBI, Harvey-Bradshaw Disease Activity Index; 5-ASA, 5-aminosalicylic acid; COVID-19, coronavirus disease 2019.*

*P < 0.10; **P < 0.05.

With respect to depression, worsening of symptoms (OR: 4.92 [95% CI: 1.58–15.29], *P* = 0.006), isolation (OR: 5.75 [95% CI: 1.69–19.59], *P* = 0.005), and drug withdrawn (OR: 2.66 [95% CI: 1.12–6.31], *P* = 0.026) were identified to be independent factors for developing depression. Likewise, telemedicine (OR: 0.13 [95% CI: 0.05–0.32], *P* < 0.001) was negatively related to depression (Table 5).

**TABLE 5:** Factors associated with elevated depression.

Variables	Univariable analysis			Multivariable analysis		
	*P*	OR	95% CI	*P*	OR	95% CI
Gender (female)	0.063*	0.51	0.25–1.04	0.162	0.53	0.22–1.29
Age	0.314	0.99	0.96–1.01			
Marital status						
Married						
Single	0.823	1.10	0.51–2.34			
Divorced	0.457	0.44	0.05–3.88			
Occupation						
Manual worker						
Mental worker	0.193	1.86	0.73–4.72			
Unemployed	0.433	0.61	0.18–2.11			
Retired	0.635	0.70	0.16–3.10			
Student	0.932	1.05	0.33–3.39			
Education level						
Elementary						
High school	0.633	1.48	0.30–7.35			
Bachelor	0.115	3.75	0.73–19.39			
Post graduate	0.112	6.75	0.64–71.17			
Socio-economic status						
Low						
Middle	0.183	1.79	0.76–4.19			
High	0.663	1.20	0.52–2.78			
Medical insurance coverage	0.293	1.64	0.65–4.15			
Smoking	0.724	0.85	0.35–2.10			
Drinking	0.209	1.90	0.70–5.17			
Disease duration	0.667	1.02	0.95–1.09			
Disease activity (HBI)	0.006*	1.22	1.06–1.40	0.859	0.98	0.80–1.21
Perianal involvement	0.400	1.34	0.68–2.66			
History of surgery	0.165	1.75	0.79–3.86			
Treatment						
5-ASA	0.456	0.71	0.29–1.73			
Prednisolone	0.999	0	/			
Immunomodulators	0.110	1.77	0.88–3.56			
Biologics	0.312	1.43	0.72–2.84			
Traditional Chinese medicine	0.941	1.10	0.10–12.39			
No medication	0.325	0.52	0.14–1.92			
Isolation	0.001*	5.20	1.92–14.09	0.005**	5.75	1.69–19.59
Use of telemedicine	<0.001*	0.19	0.09–0.40	<0.001**	0.13	0.05–0.32
Vaccination	0.200	1.61	0.78–3.33			
Knowledge of the Delta variant	0.277	1.15	0.89–1.49			
Drug withdrawn	0.006*	2.68	1.33–5.41	0.026**	2.66	1.12–6.31
Worsening of symptoms	<0.001*	4.92	2.32–10.45	0.006**	4.92	1.58–15.29
Fear of contracting COVID-19	0.508	1.26	0.63–2.53			
Stopped working	0.101	2.10	0.87–5.12			

*OR, odds ratio; CI, confidence interval; HBI, Harvey-Bradshaw Disease Activity Index; 5-ASA, 5-aminosalicylic acid; COVID-19, coronavirus disease 2019.*

*P < 0.10; **P < 0.05.

Finally, we found that no medication (OR: 7.73 [95% CI: 1.65–36.27], *P* = 0.010) and vaccination (OR: 3.07 [95% CI: 1.19–7.93], *P* = 0.021) were relevant to better life quality while worsening of symptoms (OR: 0.09 [95% CI: 0.01–0.83], *P* = 0.034) was an independent risk factor for impaired life quality (Table 6).

**TABLE 6:** Factors associated with better life quality.

Variables	Univariable analysis			Multivariable analysis		
	*P*	OR	95% CI	*P*	OR	95% CI
Gender (female)	0.891	0.95	0.42–2.11			
Age	0.115	0.97	0.94–1.01			
Marital status, *n* (%)						
Married						
Single	0.034*	2.49	1.07–5.80	0.091	2.42	0.87–6.73
Divorced	0.925	1.11	1.22–10.16	0.780	1.41	0.13–15.75
Occupation						
Manual worker						
Mental worker	0.950	1.04	0.34–3.13			
Unemployed	0.736	0.79	0.20–3.15			
Retired	0.714	0.72	0.13–4.12			
Student	0.364	1.78	0.51–6.23			
Education level						
Elementary						
High school	0.350	2.74	0.33–22.70			
Bachelor	0.396	2.57	0.29–22.80			
Post graduate	0.999	0	/			
Socio-economic status						
Low						
Middle	0.326	0.56	0.17–1.80			
High	0.691	1.21	0.48–3.02			
Medical insurance coverage	0.952	0.97	0.36–2.64			
Smoking	0.191	1.87	0.73–4.80			
Drinking	0.357	1.69	0.55–5.18			
Disease duration	0.766	1.01	0.93–1.10			
Disease activity (HBI)	0.001*	0.59	0.43–0.81	0.069	0.73	0.51–1.03
Perianal involvement	0.444	1.37	0.61–3.04			
History of surgery	0.199	0.48	0.15–1.48			
Treatment						
5-ASA	0.131	0.38	0.11–1.34			
Prednisolone	0.554	2.09	0.18–23.80			
Immunomodulators	0.667	1.20	0.53–2.71			
Biologics	0.493	0.76	0.34–1.68			
Traditional Chinese medicine	0.554	2.09	0.18–23.80			
No medication	0.044*	3.17	1.03–9.73	0.010**	7.73	1.65–36.27
Isolation	0.597	0.69	1.19–2.54			
Use of telemedicine	0.094*	2.08	0.88–4.91	0.166	2.00	0.75–5.32
Vaccination	0.042*	2.32	1.03–5.24	0.021**	3.07	1.19–7.93
Knowledge of the Delta variant	0.303	1.18	0.86–1.60			
Drug withdrawn	0.523	0.77	0.34–1.74			
Worsening of symptoms	0.008*	0.06	0.01–0.49	0.034**	0.09	0.01–0.83
Fear of contracting COVID-19	0.104	0.48	0.20–1.16			
Stopped working	0.693	0.79	0.25–2.52			

*OR, odds ratio; CI, confidence interval; HBI, Harvey-Bradshaw Disease Activity Index; 5-ASA, 5-aminosalicylic acid; COVID-19, coronavirus disease 2019.*

*P < 0.10; **P < 0.05.

## Discussion

In the current context of SARS-CoV-2 Delta variant predominance, patients suffering from CD have been forced to face dramatically altered psychological stress and life quality. In this study, we aimed to evaluate the level of anxiety and depression as well as HRQoL among CD patients and tried to explore potent risk factors, so as to optimize the management of patients with CD. As far as we know, this is the first study that focused on the impact of SARS-CoV-2 Delta variant on the mental health and life quality among the CD population. According to the analysis of our questionnaire, we found that quite a number of CD patients were confronted with different levels of anxiety and depression, with incidence of 28.10 and 31.37% for anxiety and depression, respectively. This result went in line with a previous conducted survey (29). Also, compared with non-pandemic circumstances, the life quality of CD patients due to the present situation was more often compromised (35).

Though we expected fear of contracting SARS-CoV-2 Delta variant to be a risk factor for psychological stress, no relevance was observed in our analysis. This could possibly be explained by the relatively extensive publicity on the knowledge of COVID-19, which was reflected by the high score on the perception of SARS-CoV-2 Delta variant. Also, the strictly limited traffic by the lockdown was thought to protect the citizens from contracting COVID-19, which might enhance the sense of security among CD patients.

Our findings demonstrated that isolation was closely related to both anxiety and depression. In the context of the epidemic, residents at a high risk of getting infected with COVID-19 (especially those having contacted with COVID-19 patients) were usually isolated, which on one hand stopped the patients from getting healthcare including going to the clinic, purchasing drugs, and maintaining biologics infusion and on the other hand, separated the patients from their loved ones. Hence, it is not surprising that isolation is an independent risk factor for anxiety/depression. In our study, 13.07% participants were isolated, which is in consistence with previous studies (29, 35, 36). These results implicated the necessity of offering timely psychological counseling to this high-risk population. Tremendous studies have shown that various psychological therapies could relieve psychological stress as well as alleviate disease activity in IBD patients (37, 38). In terms of this issue, many cities in China have opened psychological counseling hotlines during the epidemic, but only a small proportion of the patients are aware of and are willing to turn to it for help (21, 13.73%), suggesting that more attention need to be paid to the publicity of the psychological counseling hotlines.

In our study, worsening of symptoms showed significant relationships with depression and impaired HRQoL, which once again proved the bi-directional interaction of the brain-gut axis model (39). Growing evidence illustrated that depressive symptoms were strictly related to disease recurrence in IBD patients (40). Furthermore, higher psychological stress is verified as a predictor of lower life quality (41). These investigations together with our study remind our gastroenterologists of the importance of paying more attention to those patients with exacerbated symptoms and further addressing the knowledge of how to manage CD during pandemic in this setting of patients.

The clinical course of CD is characterized by periods of remissions with recurrent episodes, making it extremely crucial for the patients to adhere to continuous and long-term use of medication. In the present study, 64 participants (41.83%) were forced to discontinue their CD medications, among which the biologics infusion was the most affected. Our analysis showed that drug withdrawn was relevant to the occurrence of depression, which was in line with our expectation. It is widely acknowledged that 5-ASA and biologics are critic to CD treatment, and unreasonable withdrawn would trigger disease relapse (42). Hence, we recommend that CD patients should avoid drug discontinuation whenever possible. Good news is that to handle this issue, alternative options like drug delivery by hospitals have been provided, which could to a large extent solve the problem of drug withdrawn. For those on biologic treatment, we recommend that a subcutaneous dosage form such as adalimumab could be considered to replace intravenous infusion.

Apart from limited accessibility to CD drugs, many patients were also in face of difficulty in keeping in a regular contact with their treating physicians. Previous research showed that patients who talked to their healthcare providers felt more supported compared to those who did not, and they were at a lower risk of experiencing worsening of IBD symptoms, highlighting the importance of regular communication between patients and their gastroenterologists (43). To minimize the spread of SARS-CoV-2, IBD physicians in our institute started to make greater use of telehealth service, which turned out to be received with high satisfaction by CD patients. In this study, the analysis revealed that those staying in contact with the gastroenterologists by telemedicine tend to have less anxiety/depression, which partially proved that the telehealth service we provided was a nice try and was worth promoting among CD patients in isolation. The definite role of telemedicine among CD patients during the COVID-19 pandemic needs to be verified in larger cohorts and the procedure awaits standardization, but we believe that in the near future it will become an indispensable alternative for more CD patients.

Since the outbreak of the COVID-19 pandemic, tremendous efforts have been made into the research and development of effective vaccines. Up to now, several mRNA vaccines and inactivated vaccines have been approved for use in many countries and additional novel vaccines are emerging (44). It is well acknowledged that immune dysfunction is a key part during the onset of IBD and in IBD patients, the immune capacity is often compromised because of the application of immune-modifying treatment such as corticosteroids, immunomodulators and biologic agents [e.g., monoclonal antibodies for TNF-α, interleukin 12/23, integrin α4β7, and small molecules such as Janus kinase (JAK) inhibitors]. Additionally, there remain some doubts about the effectiveness of the vaccines against the Delta variant. On this basis, a number of patients are still in hesitation to get vaccinated. In our study, only 55 CD patients (35.95%) reported to have been vaccinated. Though no relevance was found between vaccination and anxiety/depression, we did observe that the vaccinated population seemed to own higher life quality. Possible explanation for this association could be that those willing to get vaccinated were in good health and therefore they were not bothered by the above-mentioned doubts. Importantly, we also detected a desire among CD patients to get protection from vaccination. In fact, several researches on the use of vaccines have been carried out in IBD patients, which confirmed the safety and efficacy of the vaccines among this specific population (45–47). According to recommendations from International Organization for the Study of Inflammatory Bowel Disease (IOIBD), patients with IBD should be vaccinated against SARS-CoV-2 as early as possible, and vaccination should not be delayed due to the ongoing immunoregulatory therapy (48). Also, the already existed vaccines remain effective against the Delta variant (49). Hence, to relieve the anxiety and enhance immune defense of CD patients during the COVID-19 pandemic, we need to reassure our CD patients of SARS-CoV-2 vaccination.

The main limitations of this study mainly consisted of the relatively small sample because of the online property of the procedure. A total of 153 participants were recruited, which only accounted for about 50% of all CD patients that we can obtain by the electronic medical records. Also, since the questionnaire were self-reported, subjectivity is a non-negligible factor when we turn these findings into clinical practice. In some cases, the degree of anxiety/depression might be over-exaggerated or under-estimated, and the assessment of disease severity could also be inaccurate. Additionally, those without accessibility to the internet were naturally excluded from the survey, making this population a blind spot of our study. In can be inferred that this group of patients are probably older and might live in remote places, which indicates higher psychological stress due to worse knowledge about the SARS-CoV-2 Delta variant and ambiguity in their disease severity. In addition, it is very likely that the changes in mental anxiety were associated with changes in the number of SARS-CoV-2-infected patients, which could possibly be explained by consequent alterations in the administration of the mandatory confinement of the population. However, in our study, the survey was conducted only once, so the changes in mental anxiety could not be captured. To verify this hypothesis, consecutive studies should be conducted to monitor the changes in mental anxiety among CD patients. Hence, these factors should be taken into consideration when using the results of our study. Nevertheless, our study provides first-hand information about the incidence and risk factors of anxiety/depression and impaired life quality in CD patients in the context of the SARS-CoV-2 Delta variant dominance. Further prospective longitudinal studies are necessary to validate our findings.

## Conclusion

During SARS-CoV-2 Delta variant predominance, many CD patients suffered from symptoms of anxiety and depression and impaired life quality. Those in isolation or with worsening of symptoms and drug withdrawn were more prone to experience psychological stress. Individualized management such as drug delivery and telemedicine should be promoted to maintain control of mental health and life quality during the pandemic.

## Data Availability Statement

The original contributions presented in the study are included in the article/Supplementary Material, further inquiries can be directed to the corresponding authors.

## Ethics Statement

The studies involving human participants were reviewed and approved by the Institutional Review Board and Ethics Committee of Affiliated Hospital of Yangzhou University (REC ref 2021-YKL06-09-006). The patients/participants provided their written informed consent to participate in this study. Written informed consent was obtained from the individual(s) for the publication of any potentially identifiable images or data included in this article.

## Author Contributions

JL and YS carried out the studies, participated in the experimental design and statistical analysis, and drafted the manuscript. XH and TZ participated in the analysis of the overall data. GY and WX were responsible for the second check of relevant data. YD participated in and supervised the whole study process. SH and MW critically revised important knowledge content. All authors read and approved the final manuscript.

## Conflict of Interest

The authors declare that the research was conducted in the absence of any commercial or financial relationships that could be construed as a potential conflict of interest.

## Publisher’s Note

All claims expressed in this article are solely those of the authors and do not necessarily represent those of their affiliated organizations, or those of the publisher, the editors and the reviewers. Any product that may be evaluated in this article, or claim that may be made by its manufacturer, is not guaranteed or endorsed by the publisher.

## Supplementary Material

Supplementary Figure 1 Flowchart of study process.

Supplementary Table 1 Questionnaire assessing patients’ knowledge of the COVID-19 pandemic and the SARS-CoV-2 Delta variant.

## Abbreviations

COVID-19: coronavirus disease 2019
CD: Crohn’s disease
HRQoL: health-related quality of life
VoCs: variants of concern
IBD: inflammatory bowel disease
ENS: enteric nervous system
SP: substance P
VIP: vasoactive intestinal peptides
TNF- α: tumor necrosis factor α
ECCO: European Crohn’s and Colitis Organization
HBI: Harvey-Bradshaw Disease Activity Index
GAD-7: Generalized Anxiety Disorder Scale 7
PHQ-9: Patient Health Questionnaire-9
IBDQ: Inflammatory Bowel Disease Questionnaire
SD: standard deviation
5-ASA: 5-aminosalicylic acid
JAK: Janus kinase
IOIBD: International Organization for the Study of Inflammatory Bowel Disease.
