# Supplementary figures and images for: Impact of the SARS-CoV-2 Delta Variant on the Psychological States and Health-Related Quality of Life in Patients With Crohn’s Disease
Source: Front Med (Lausanne). 2022 Mar 29;9:795889. doi: 10.3389/fmed.2022.795889 (PMC9001935; doi:10.3389/fmed.2022.795889)

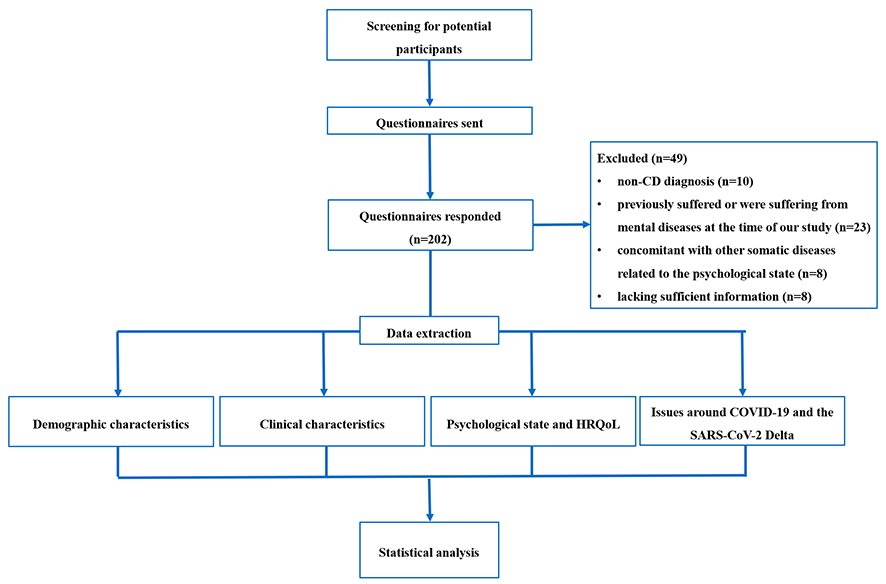

Supplement: Supplementary Figure 1 — Flowchart of study process. [file Image_1.TIF]
