# Supplementary material for: Impact of the SARS-CoV-2 Delta Variant on the Psychological States and Health-Related Quality of Life in Patients With Crohn’s Disease
Source: Front Med (Lausanne). 2022 Mar 29;9:795889. doi: 10.3389/fmed.2022.795889 (PMC9001935; doi:10.3389/fmed.2022.795889)
Supplement: Supplementary Table 1 — Questionnaire assessing patients’ knowledge of the COVID-19 pandemic and the SARS-CoV-2 Delta variant. [file Table_1.DOCX]

| Knowledge of the COVID 19 pandemic and the SARS-CoV-2 Delta variant |
| --- |
| Q1. Can wearing a mask effectively prevent the Delta variant?  A. Yes B. No |
| Q2. Which strain spreads faster, the Delta strain or the normal strain? A. Delta strain B. Normal strain |
| Q3. Is the COVID-19 vaccine effective against the Delta variant? A. Yes B. No C. I don’t know |
| Q4. Compared with the normal strain, how many times is the viral load of the Delta strain? A. 260 times B. 1260 times C. 2260 times D. 3260 times |
| Q5. It takes only 14 seconds to get infected when contacting with a carrier of the Delta mutation. A. Ture  B. False |
| Q6. No fever means you are definitely not infected with Delta variant. A. Ture  B. False |
| Q7. Symptoms of the Delta strain infection include low fever symptoms in the early stages, and many patients have fatigue, smell disorder, and mild muscle soreness. A. Ture B. False |
| Q8. The modes of transmission of the Delta strain include A. Droplet transmission B. Close contact C. Aerosol transmission D. Exposure to viral contaminants |
| Q9. Which of the following are effective ways to reduce the spread of the Delta strain (multiple choice)? A. Wear a mask B. Wash hands C. Avoid gathering D. Avoid taking public transportations |
